# Estimation of PfRh5-based vaccine efficacy in asymptomatic *Plasmodium falciparum* patients from high-endemic areas of Tanzania using genetic and antigenicity variation screening

**DOI:** 10.3389/fimmu.2024.1495513

**Published:** 2024-11-18

**Authors:** Hojong Jun, Ernest Mazigo, Wang-Jong Lee, Johnsy Mary Louis, Jadidan Hada Syahada, Fadhila Fitriana, Jin Heo, Yeonkyung Kim, Boeun Kwon, Fauzi Muh, Feng Lu, Md Atique Ahmed, Se Jin Lee, Sunghun Na, Wanjoo Chun, Won Sun Park, Min Hong, Joon-Hee Han, Tae-Hyung Kwon, Soo-Ung Lee, Eun-Teak Han, Jim Todd, Alphaxard Manjurano, Winifrida Kidima, Jin-Hee Han

**Affiliations:** ^1^ Department of Medical Environmental Biology and Tropical Medicine, School of Medicine, Kangwon National University, Chuncheon, Republic of Korea; ^2^ Department of Parasitic Diseases, National Institute for Medical Research, Dar es Salaam, Tanzania; ^3^ Faculty of Public Health, Department of Epidemiology and Tropical Diseases, Universitas Diponegoro, Semarang, Indonesia; ^4^ Department of Pathogen Biology and Immunology, School of Medicine, Yangzhou University, Yangzhou, China; ^5^ Malaria Division, Indian Council of Medical Research (ICMR)-Regional Medical Research Centre, Dibrugarh, Assam, India; ^6^ Department of Obstetrics and Gynecology, Kangwon National University Hospital, Chuncheon, Republic of Korea; ^7^ Department of Pharmacology, School of Medicine, Kangwon National University, Chuncheon, Republic of Korea; ^8^ Department of Physiology, School of Medicine, Kangwon National University, Chuncheon, Republic of Korea; ^9^ Institute of Biological Resources, Chuncheon Bioindustry Foundation, Chuncheon, Republic of Korea; ^10^ Department of Population Health, London School of Hygiene and Tropical Medicine, London, United Kingdom; ^11^ Department of Biostatistics, Catholic University of Health and Allied Sciences (CUHAS), Mwanza, Tanzania; ^12^ Department of Zoology, College of Natural and Applied Sciences, University of Dar es Salaam, Dar es Salaam, Tanzania

**Keywords:** malaria, *Plasmodium falciparum*, asymptomatic, Tanzania, PfRh5, genetic variation, antigenicity variation, vaccine

## Abstract

**Background:**

*Plasmodium falciparum* is the most lethal malaria parasite. Recent phase 1b vaccine trials using *P. falciparum* reticulocyte binding protein homolog 5 (PfRh5) demonstrated safety and promising efficacy in preventing merozoite invasion. PfRh5 has emerged as a strong vaccine candidate due to its essential role in merozoite invasion and limited sequence variation. For effective malaria vaccine development, especially in high-transmission settings, strain-transcending activity must be considered. Ongoing monitoring of antigenic variation and natural immune responses is important to estimate vaccine efficacy across geographically diverse populations.

**Methods:**

Samples for this study were collected from four villages in each of the Kigoma and Geita regions, known malaria transmission hotspots in Tanzania. This community-based cross-sectional study was conducted from December 2022 to July 2023. Genetic variation and natural selection pressure on *pfrh5* were analyzed in 164 asymptomatic *P. falciparum* isolates. The humoral immune response to PfRh5 was also assessed using a protein microarray with 242 sera samples from asymptomatic patients in the same population. Finally, a correlation analysis was conducted to compare pfrh5 genetic variation with the humoral immune response.

**Results:**

The results revealed that *pfrh5* was well conserved, but novel non-synonymous mutations were found at D65H, H170N, and I227M. Additionally, natural selection metrics indicated the potential for positive selection and a recent population expansion of PfRh5 in the study area, both of which could influence vaccine effectiveness. Antigenicity screening revealed variable sensitivity, ranging from 3.3% in Bunyambo to 82.8% in Rwantaba, with no significant relationship between antigenicity and parasitemia, haplotypes, or gender. However, age was significantly associated with humoral immune response (*ρ* = 0.170, *p* = 0.008).

**Conclusions:**

These findings underscore the need for future PfRh5-based vaccines to consider for increasing genetic variation and geographical differences in humoral immune responses.

## Introduction

Malaria continues to be a major global health concern, with approximately 249 million cases reported worldwide in 2022, a slight increase from the 244 million cases reported in 2021 (1). This infectious disease caused around 608,000 deaths in 2022, a small decrease from the 610,000 deaths reported the previous year (1). The Sub-Saharan African (SSA) regions exhibit highest burden of malaria, accounting for 94% of all cases and 95% of all deaths globally. Children under five years old are the most vulnerable, tragically accounting for approximately 78% of malaria-related deaths in these regions. Tanzania, located in Sub-Saharan Africa, was affected by 8 million malaria cases and 26,600 deaths in 2022, reflecting the ongoing severity of malaria risk in the country. The ongoing challenges in malaria controlling is emerging resistance to antimalarial drugs and insecticides (2, 3). Additionally, climate change is a new risk hindering efforts to malaria control by altering the habitats and behaviors of mosquito vectors and resulting in an increasing heterogeneity of *Plasmodium* species (4). Given the recent acceleration in the globalized world, it is essential to continuously monitor the increasingly diverse risks associated with malaria transmission patterns (5). In response to these challenges, vaccination has become a most important strategy in controlling malaria risk.

Recently, the RTS, S/AS01 (Mosquirix™) vaccine has been approved by the World Health Organization (WHO). The RTS, S-based vaccine is a pre-erythrocytic vaccine that targets the circumsporozoite protein (CSP) on the surface of sporozoites and aims to prevent the infection of hepatocytes (6). This vaccine has been shown malaria protection rate by approximately 30% in children who complete the full vaccination schedule of four doses in field tests (7). The second malaria vaccine approved by the WHO is R21/Matrix-M. This vaccine was prequalified by the WHO in 2023 after successful clinical trials demonstrated its sufficient efficacy and safety, especially in children. The R21/Matrix-M vaccine showed a protection rate of more than 75% in clinical trials, representing a significant improvement over the first approved malaria vaccine RTS, S/AS01 (8). It includes Hepatitis B antigen fused to the C-terminus and central repeats of the CSP (Asn-Ala-Asn-Pro), which self-assemble into virus-like particles in yeast (9). The major target of R21/Matrix-M is CSP, which classifies it as a pre-erythrocytic vaccine. Although these two vaccines provide a great opportunity for malaria protection, it is important to consider the genetic variation across a wide range of field strains. Both vaccines target CSP, which is a sporozoite surface protein that generally exhibits significant genetic variation due to host immune evasion or adaptation to interactions with host cells (10, 11). Thus, it is necessary to identify conserved antigens to develop an effective vaccine across diverse strains.

Three strategies are in the pipeline for malaria vaccine development, including pre-erythrocytic stage blocking, transmission blocking, and blood-stage blocking approaches (12). Among them, the blood-stage vaccine focuses on reducing malaria risk by blocking merozoite invasion into erythrocytes at the individual level, thereby reducing morbidity and mortality in malaria-endemic regions (13). The functional importance of a blood-stage vaccine also includes reducing heavy infection in patients in malaria-endemic areas, including asymptomatic cases, which leads to further decreases in transmission (14).

One promising target antigen for a blood-stage vaccine is *P. falciparum* reticulocyte-binding protein homolog 5 (PfRh5). Unlike other invasion ligands, PfRh5 is essential for invasion across various strains and is considered one of the most promising candidates for a blood-stage vaccine (15). PfRh5 forms a complex with proteins cysteine-rich protective antigen (CyRPA) and Rh5 interacting protein (RIPR) (RCR complex) to bind to the erythrocyte surface receptor basigin (CD147), which is a critical step in the merozoite invasion process (16). Research has demonstrated that antibodies against PfRh5 can effectively block merozoite invasion, leading to multiple clinical trials exploring vaccines based on PfRh5 (17, 18). The Rh5.1 soluble protein and PfRh5 viral-vectored vaccine used full-length of the antigen are in the phase 1b clinical trials (19, 20). These clinical studies in Tanzania demonstrated acceptable safety and elicited humoral immune responses in volunteers, showing up to 50-60% invasion inhibition activity in laboratory strains (19, 20).

However, a key challenge in developing PfRh5-based vaccines is ensuring a robust and long-lasting immune response that remains effective against a wide range of field-isolated parasite strains. To address this, it is essential to continuously monitor the genetic polymorphisms and antigenicity in patients across different regions to evaluate and ensure the ongoing efficacy of the vaccine (21). Recent studies have introduced a platform for evaluating malaria vaccine candidates, which can help assess key considerations for the development and maintenance of effective vaccines (22, 23). Thus, this study focuses on PfRh5 genetic polymorphism and patient antigenicity across different geographical regions to estimate vaccine efficacy in high-endemic settings in Tanzania. Additionally, this study used clinical isolates from asymptomatic patients to compare genetic variation and antigenicity to evaluate the development of effective PfRh5-based blood-stage vaccines.

## Methods

### Study site selection

**Figure 1:**
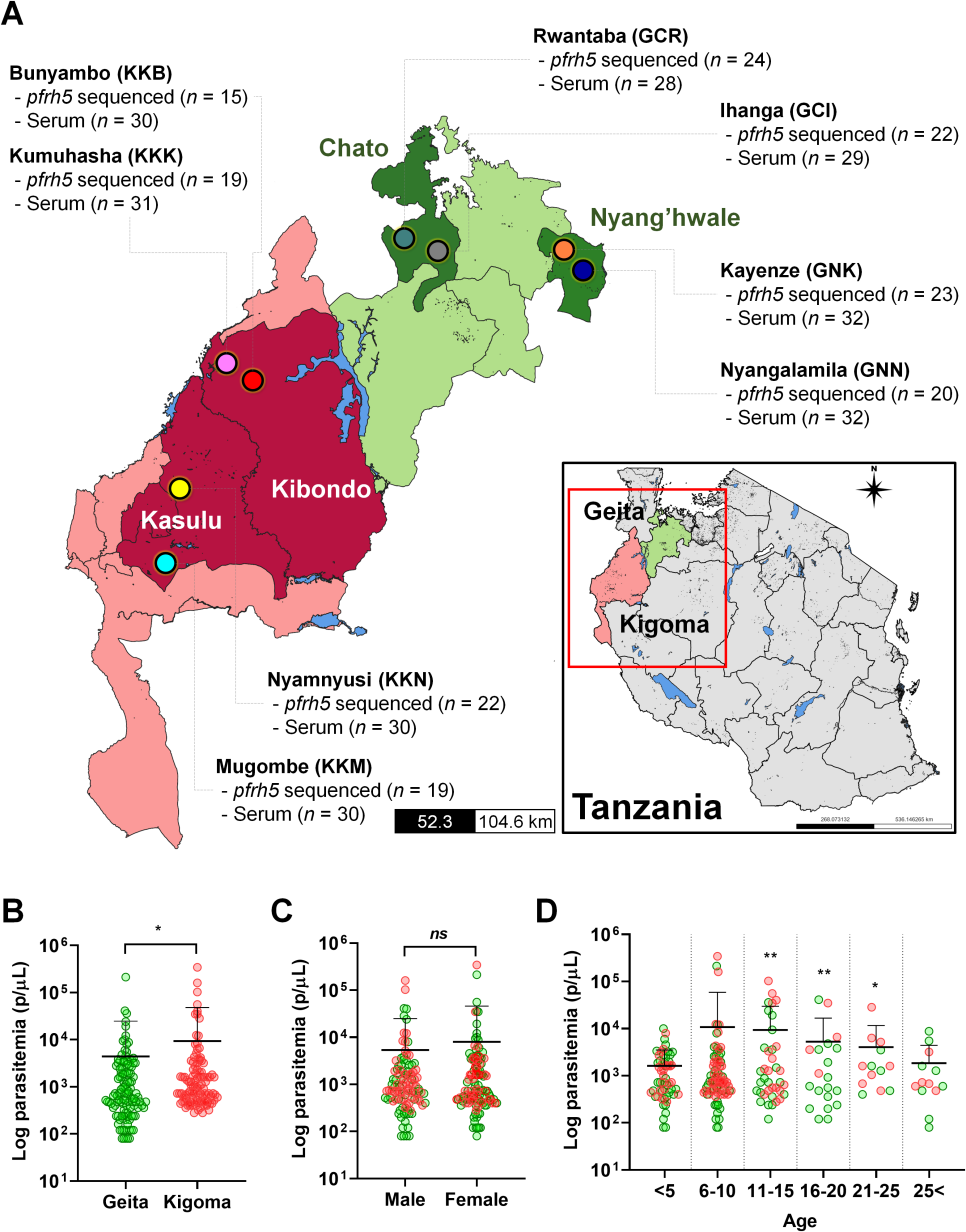
The study site and baseline information of *P. falciparum* asymptomatic patients. **(A)** The map of Tanzania shows the regions (Geita and Kigoma), districts, and villages involved in the study. Clinical isolates were collected from asymptomatic patients in eight villages, each with a varying number of patients, for PfRh5 genetic variation (*pfrh5* sequenced *n* = number of *pfrh5* successfully sequenced) and antigenicity analysis (Serum *n* = total number of participants used for antigenicity screening). **(B)** The log parasitemia (parasites/µL, p/µL) differed significantly between the studied regions, Geita and Kigoma (*p* = 0.0179). **(C)** The log parasitemia (p/µL) was not significantly different to gender of participants. **(D)** Comparison of log parasitemia (p/µL) across age groups revealed a significant difference in the 11–25 years old group compared to the <5 years old group. These differences are denoted by a double asterisk (*p* < 0.01), a single asterisk (*p* < 0.05), and ns (not significant) by an unpaired student's *t*-test. Green dots indicate isolates from Geita region, and the red dots indicate isolates from Kigoma region.

The samples for this study were collected in a community-based cross-sectional study conducted from December 2022 to July 2023. Two regions, Geita and Kigoma, were purposefully chosen due to their high malaria transmission rates in Tanzania. In 2022, malaria prevalence in both regions was 13%. From each region, two districts were randomly selected (Geita; Nyang’hwale and Chato, Kigoma; Kibondo and Kasulu), and from each district, two villages were randomly chosen (**Figure 1**). In total, the study covered eight villages across Tanzania’s high-endemic areas for *P. falciparum*.

### Clinical isolates collection and ethics statement

A research team consisting of a medical doctor and laboratory technician visited house-to house and collected whole blood samples from asymptomatic *P. falciparum* participants. After obtaining consent, a structured questionnaire was used to gather demographic information from participants. Asymptomatic cases were defined as individuals with a positive microscopic examination for any level of parasitemia, who had been afebrile, showed no malaria symptoms in the past 4-5 days, and had not used antimalarial medication in the past 7 days. Immediately after collection, blood was tested for malaria infections using the Bioline™ Malaria Ag *P.f/Pan* rapid diagnostic test (RDT) (Abbott, Chicago, IL, USA). Additionally, thin blood smears were prepared and stained with Giemsa for examination under a light microscope by two experienced microscopists. Parasitemia was counted according to the WHO Malaria Microscopy Standard Operating Procedure, based on the observation of 200 white blood cells. Patients identified as asymptomatic cases with positive *P. falciparum* tests were treated with artemether-lumefantrine in accordance with Tanzania’s malaria treatment policy. The specimens were then processed, with whole blood preserved as dried blood spots (DBS) on Whatman 3MM filter paper and serum separated and stored in a laboratory near the collection site. Serum from asymptomatic patients was kept at -20°C until use. The DBS and serum were subsequently transported to Kangwon National University for molecular diagnosis, PfRh5 sequencing, and antigenicity screening. A total of 242 whole blood samples were collected, and 242 serum (100%) were separated for antigenicity screening. However, due to the low quality of DBS and low parasitemia, only 164 isolates (67.8%) were successfully analyzed for the full-length of *pfrh5* sequence. Healthy sera (*n* = 56) for the protein array negative control were obtained from individuals under the age of 10 living in malaria non-endemic areas of the Republic of Korea during general health check-ups at Kangwon National University Hospital.

All clinical samples were collected following ethical guidelines and approved protocols from the National Institute for Medical Research (NIMR), a division of the Ministry of Health (MoH) in Tanzania (NIMR/HQ/R.81/Vol.IX/4114). The protocol for clinical samples handling was also approved by the Ethical Review Board at Kangwon National University (KWNUIRB-2022-06-008). Informed written consent was obtained from all adult participants, while for child participants, consent was secured from a parent or guardian.

### Genomic DNA extraction, target gene amplification, and sequencing

Genomic DNA was extracted from 50 µL of dried blood spots (one spot) using the QIAamp DNA Mini Kit (QIAGEN, Hilden, Germany), following the manufacturer’s protocol, with a final elution volume of 50 µL. A total of 164 isolates from Tanzania were processed to amplify and sequence the *pfrh5* gene. PCR was performed to amplify the target gene for sequencing and gene variation analysis. To cover the entire *pfrh5* gene sequence, the sequencing primers were designed for three regions (**Supplementary Table S1**). The PCR mixture, prepared in a total volume of 20 μL, contained KOD one PCR Master mix (Toyobo, Osaka, Japan), 1 μL of template DNA (gDNA from asymptomatic patients), 16 μL of distilled deionized water (DDW), and 1.0 μL of each 10 μM primer. The PCR conditions included an initial denaturation at 98°C for 7 minutes, followed by 45 cycles of 98°C for 10 seconds, 58°C for 5 seconds, and 68°C for 10 seconds, with a final extension at 68°C for 10 minutes. The amplicons were purified and visualized using electrophoresis with 1.2% agarose gel. The purified fragments were then subjected to Sanger sequencing (Nbit, Chuncheon, Republic of Korea) using sequencing primers (**Supplementary Table S1**). The sequences from each isolate were confirmed for quality by chromatogram analysis and assembled using SnapGene software version 2.3.2 and the Lasergene package to obtain the complete *pfrh5* gene sequence. The low-frequency genetic mutations were confirmed by performing three independent experiments. The detailed whole gene sequences of *pfrh5* are available at GenBank (accession numbers: PQ337615 - PQ337778).

### Nucleotide diversity and natural selection pressure

*Pfrh5* nucleotide diversity (*π*) measures the average number of nucleotide differences per site between two sequences. The number of polymorphic sites, haplotypes, and haplotype diversity (Hd) were calculated using DnaSP software version 5.10 to evaluate sequence diversity within the group (24, 25). To investigate evidence of natural selection (non-random evolution) within the species, we applied Tajima’s D, Fu and Li’s D* and F*, and Fu’s Fs tests. Under neutral evolution, Tajima’s D is expected to be zero. Significant positive values of Tajima’s D may suggest deviations from neutral mutation, potentially reflecting population contraction or balancing selection, while significant negative values could indicate recent population expansion following a bottleneck or a recent selective sweep (26). Fu and Li’s D* and F* tests, with significant positive values, generally point to population contraction due to natural selection pressure, whereas negative values often suggest population expansion and an excess of singletons (27). Fu’s Fs test evaluates allele or haplotype distribution, with negative values indicating an excess of alleles, which may suggest recent population expansion or genetic hitchhiking (28). Furthermore, Nei and Gojobori’s method was used to calculate the proportion of synonymous substitutions per synonymous site (d_S_) and non-synonymous substitutions per non-synonymous site (d_N_), with 1,000 bootstrap replications in MEGA 11 software. A d_N_-d_S_ greater than zero typically indicates positive selection, while a ratio less than one suggests purifying selection. The McDonald-Kreitman (MK) test was used to compare within-species variation to divergence between species to assess neutrality. In this test, *P. reichenowi rh5* (PRCDC_0421300), which is a species in Laverania clade that related to *P. falciparum*, was used as an out-group species and the analysis was performed with DnaSP software. Haplotype diversity of *Pfrh5* was analyzed using DnaSP, and clustering patterns were visualized using the median-joining method in Network 10.0 software.

### Tertiary structure modelling and visualization

The tertiary structure of PfRh5 was obtained from the Protein Data Bank (PDB, https://www.rcsb.org/) using IDs 8CDD and 4u0q. The 8CDD structure represents the PfRh5-PfCyRPA-PfRipr complex, while the 4u0q structure contains the PfRh5-Basigin binding complex. Both tertiary structures were aligned using ClustalW based on their sequences by PyMol, and a predicted model of the Basigin-PfRh5-PfCyRPA complex was generated. The structure was then visualized in PyMol to highlight the positions of non-synonymous mutations, particularly on the interaction surface of PfRh5 residues.

### Recombinant protein expression

The recombinant PfRh5-ecto construct was designed based on the 3D7 strain sequence (PF3D7_0424100) and included amino acids 25-526 with an expected molecular weight of 64.4 kDa including the tag. This construct comprises all non-synonymous mutation residues necessary for antigenicity screening. The protein was produced using the HEK293 EBNA1-6E cell-based expression system (29). To generate the protein, the codon-optimized *pfrh5* gene was amplified with a specific primer set (**Supplementary Table S1**) and subcloned into the expression vector pTT5-8x His using the In-Fusion^®^ HD Cloning Kit (Clontech, Mountain View, CA, USA). The final construct was then transfected into HEK293 EBNA1-6E cells using transfection grade linear polyethylenimine hydrochloride. After five days for transfection, the secreted proteins were collected from the culture supernatant and purified using Ni-NTA agarose (QIAGEN) with elution buffer (350 mM imidazole, 50 mM HEPES, 5% glycerol, 150 mM NaCl). The purified recombinant protein (1 µg) was prepared with 2x reducing buffer and separated by 4-12% gradient Bis-Tris Mini Protein Gel (Invitrogen, Waltham, MA, USA). After electrophoresis, the Bis-Tris gels were stained with Coomassie Brilliant Blue (Sigma-Aldrich).

### Antigenicity screening

Protein microarray analysis was performed to assess the antigenic variation of PfRh5. Three aminopropyl-coated slides were prepared as described previously (30). Each slide was printed with PfRh5-ecto at an optimized concentration of 50 ng/µL per spot and incubated for 2 hours at 37°C. After incubation, the slides were blocked with a solution of 5% BSA in PBS-T (0.1% Tween 20) for 1 hour at 37°C. Sera from healthy individuals and asymptomatic *P. falciparum* patients were diluted 1:50 in PBS-T and applied to each spot in duplicate on the slides for 1 hour at 37°C. The arrays were then visualized with 10 ng/µL of Alexa Fluor 546 goat anti-human IgG (Invitrogen) in PBS-T for 1 hour at 37°C, followed by scanning with the InnoScan 300 (INNOPSYS, Carbonne, France). The positive cut-off values were determined by adding two standard deviations to the average mean fluorescence intensity (MFI) of healthy sera, which were used as a negative control.

### Statistical analysis

The antigenicity screening results were analyzed and visualized using GraphPad Prism v8 (GraphPad Software, San Diego, CA, USA) and SigmaPlot v12.0 (Systat Software Inc., San Jose, CA, USA) software. For the protein array data, an unpaired Student’s *t*-test was used to compare the measured values between groups. Pearson correlation (*ρ*) analysis was applied to evaluate the relationship between antigenicity and clinical factors such as age and parasitemia (parasites/μL). Significant differences were considered as *p* < 0.05 at 95% CI.

## Results

### Study site and sample size

This study collected *P. falciparum* isolates from asymptomatic participants in Geita and Kigoma regions in Tanzania, among of the regions showed high *P. falciparum* prevalence in 2022. From each region, two districts were randomly selected followed by a random selection of two villages from each district. In Geita region, Chato and Nyang’hwale districts were chosen. From Chato district, selected villages were Rwantaba (GCR) and Ihanga (GCI) whereas Kayenze (GNK) and Nyangalamila (GNN) were respectively chosen from Nyang’hwale district. In Kigoma region, Kasulu and Kibondo districts were selected. From Kasulu district, the villages chosen were Mugombe (KKM) and Nyamnyusi (KKN), while from Kibondo district, the selected villages were Bunyambo (KKB) and Kumuhasha (KKK) (**Figure 1A**).

Baseline information and parasitemia from asymptomatic participants who were positive for *P. falciparum* were compared with demographic factors (**Supplementary Table S2**). Significant differences in parasitemia were observed between regions, with Geita showing a mean parasitemia of 4,400 ± 20,343 parasites/µL and Kigoma showing 9,381 ± 38,880 parasites/µL (**Figure 1B**). However, there was no significant differences between parasitemia and gender (**Figure 1C**). When comparing by age groups, younger than 5 years old exhibited lower parasitemia levels, whereas those aged in 11 to 25 years showed significantly higher parasitemia compared to the younger age group (<5 years old) (**Figure 1D**).

### *PfRh5* gene sequence characteristics and 3D structure prediction

**Figure 2:**
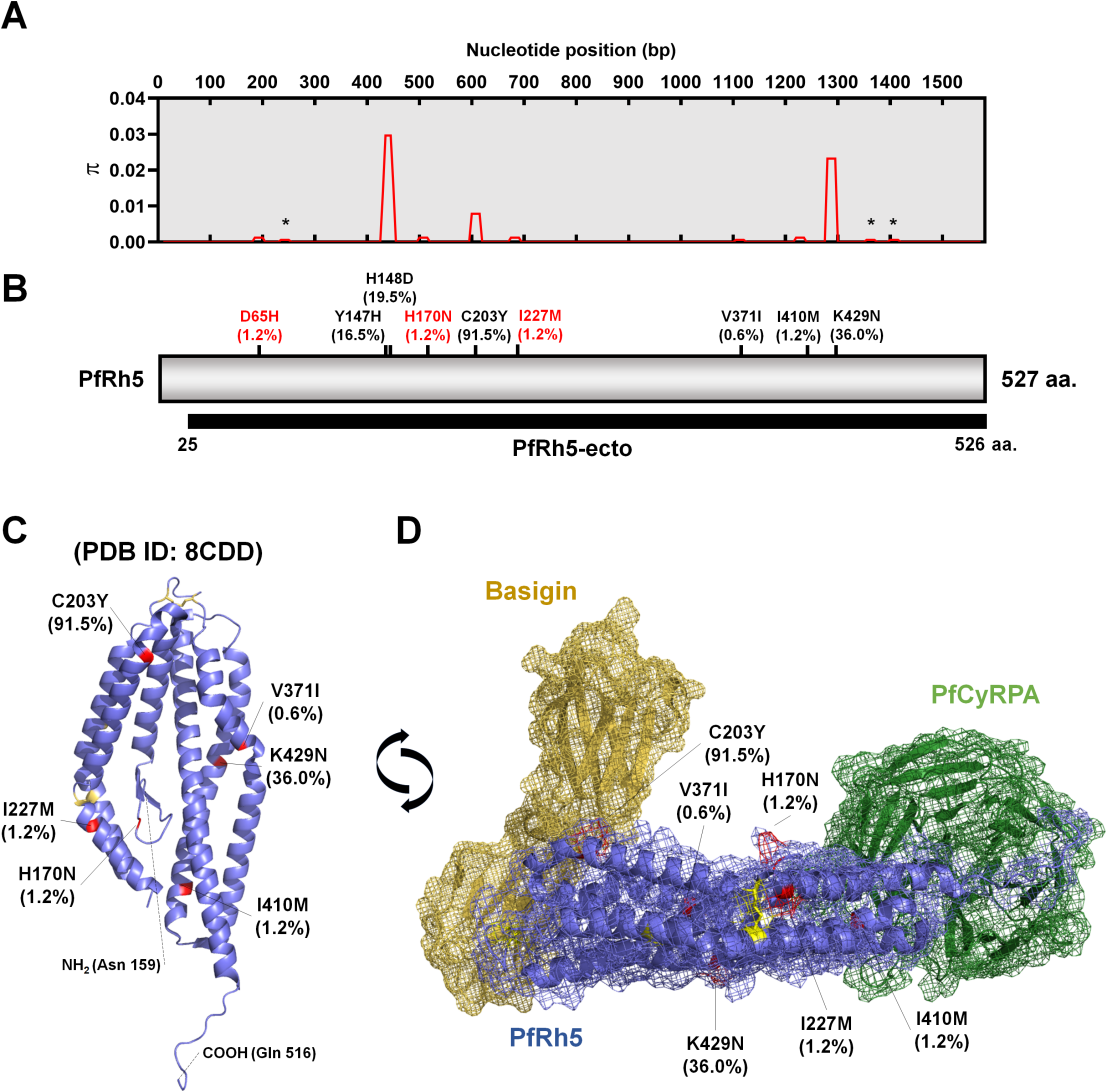
Schematic diagram of PfRh5 genetic diversity and protein tertiary structure. **(A)** Presentation of nucleotide diversity (π), based on nucleotide positions, indicating limited polymorphic sites within studied areas. The star mark represents a synonymous mutation site. **(B)** Amino acid-based schematic structure of PfRh5 highlighting frequency of non-synonymous mutations and their varying proportions within the isolates. The red-colored mutation sites indicate the novel identified non-synonymous mutations. The black bar indicates the recombinant protein expression domain (PfRh5-ecto; 25–526 aa.) used for antigenicity analysis, which covers all non-synonymous mutation residues. **(C)** Protein structure (PDB ID: 8CDD) highlighting each non-synonymous mutation in red. Yellow-colored residues represent disulfide bridges within PfRh5. **(D)** The interaction model of PfRh5 with Basigin (PDB ID: 4u0q) and PfCyRPA (PDB ID: 8CDD) highlighting the mutation sites.

The complete *pfrh5* (PF3D7_0424100) genomic DNA sequence spans 1,788 base pairs and is located on chromosome 4. It comprises one intron and two exons, with the coding sequence in total 1,581 base pairs (527 amino acids) and a predicted molecular weight of 62.9 kDa (**Figures 2A, B**). Nucleotide diversity (*π*) among clinical isolates showed limited variation in codons (**Figure 2A**). Most mutations were non-synonymous, with Y147H (16.5%), H148D (19.5%), C203Y (91.5%), and K429N (36.0%) being relatively high in prevalence (**Figure 2B**). Non-synonymous mutations with low frequencies were observed at V371I (0.6%) and I410M (1.2%). Additionally, novel mutations were identified at D65H (1.2%), H170N (1.2%), and I227M (1.2%), both of which occurred at low frequencies (**Figure 2B**).

The protein tertiary structure, obtained from PDB (ID: 8CDD), represents the PfRh5-PfCyRPA-PfRipr (RCR) complex and highlights the non-synonymous mutation residues within the PfRh5 structure (159-516 aa.) (**Figure 2C**). PfRh5 directly interacts with PfCyRPA, while another region of PfRh5 binds with Basigin (CD147) as a host cell receptor. We also obtained the PfRh5-Basigin complex structure from the PDB (ID: 4u0q) and reconstructed the Basigin-PfRh5-PfCyRPA complex (**Figure 2D**). The amino acid residues involved in the interaction with PfCyRPA and Basigin were well conserved (**Figure 2D**).

### Genetic diversity and natural selection pressure of *pfrh5*

Among the 164 isolates from eight villages, we identified 12 polymorphic sites in *pfrh5*, comprising 3 synonymous and 9 non-synonymous sites. The highest nucleotide diversity (*π*) was observed in Kibondo district (*π* ± SD., 0.00105 ± 0.00011), with Kumuhasha (KKK, 0.00109 ± 0.00009), and Bunyambo (KKB, 0.00098 ± 0.00021) (**Table 1**; **Supplementary Figure S1A**). Following Kibondo district, Kasulu district (0.00082 ± 0.00012) showed high nucleotide diversity in Mugombe (KKM, 0.00099 ± 0.00020), with the lowest diversity observed in Nyamnyusi (KKN, 0.00070 ± 0.00016) (**Table 1**; **Supplementary Figure S1A**). Comparatively, Geita region (0.00080 ± 0.00007) showed lower nucleotide diversity than Kigoma region (0.00093 ± 0.00009) (**Table 1**; **Supplementary Figure S1A**). Rwantaba village (GCR, 0.00086 ± 0.00013) in Chato district exhibited the highest nucleotide diversity within Geita region. This was followed by Kayenze (GNK, 0.00080 ± 0.00016), Nyangalamila (GNN, 0.00079 ± 0.00012), and Ihanga (GCI, 0.00073 ± 0.00016) (**Table 1**; **Supplementary Figure S1A**). Overall, the population-wide nucleotide diversity was 0.00086 ± 0.00006 (π ± S.D.) in the study areas (**Table 1**; **Supplementary Figure S1A**).

**Table 1:** Estimates of nucleotide diversity, haplotype diversity and neutrality indices of *pfrh5* based on the geographical location in Tanzania.

Location	No. of samples	SNPs	No. of haplotype	Diversity ± S.D.		Tajima's D	Fu and Li's	
				Haplotype (Hd)	Nucleotide (*π*) X 10^-3^		D*	F*
**Geita**	**89**	**8**	**12**	**0.713 ± 0.043**	**0.80 ± 0.07**	**-0.49990**	**-1.90233**	**-1.68816**
Chato	46	6	10	0.722 ± 0.056	0.79 ± 0.10	-0.21442	-0.51159	-0.49020
GCI	22	6	7	0.684 ± 0.097	0.73 ± 0.16	-0.93904	-0.91740	-1.06918
GCR	24	4	8	0.722 ± 0.065	0.86 ± 0.13	0.73416	1.08443	1.13903
Nyang’hwale	43	6	9	0.695 ± 0.063	0.80 ± 0.10	-0.22701	-0.48487	-0.47358
GNK	23	6	7	0.704 ± 0.089	0.80 ± 0.16	-0.69414	-0.21852	-0.41180
GNN	20	4	6	0.705 ± 0.086	0.79 ± 0.12	0.29432	0.17445	0.23919
**Kigoma**	**75**	**9**	**11**	**0.773 ± 0.031**	**0.93 ± 0.09**	**-0.51652**	**-0.09573**	**-0.28156**
Kibondo	34	6	7	0.802 ± 0.038	1.05 ± 0.11	0.35240	0.40781	0.45624
KKB	15	5	6	0.762 ± 0.096	0.98 ± 0.21	0.01126	0.48090	0.40698
KKK	19	5	6	0.766 ± 0.072	1.09 ± 0.09	0.65310	0.40275	0.54394
Kasulu	41	8	10	0.740 ± 0.053	0.82 ± 0.12	-0.86846	-0.74055	-0.91607
KKM	19	8	9	0.778 ± 0.096	0.99 ± 0.20	-1.07839	-0.32512	-0.62127
KKN	22	4	5	0.684 ± 0.063	0.70 ± 0.16	0.00584	1.09548	0.91166
**Overall**	**164**	**12**	**17**	**0.745 ± 0.026**	**0.86 ± 0.06**	**-0.88994**	**-1.27193**	**-1.35296**

The bold text indicates the region level.

To investigate whether allele frequencies at the *pfrh5* polymorphisms reflect evidence of selection rather than random mutation, several tests were conducted at both intra- and inter-species levels. Among the intra-species tests, Tajima’s D (-0.88994) indicates an excess of low-frequency alleles, suggesting recent population expansion. Similarly, the negative values for Fu and Li’s D* (-1.27193) and Fu and Li’s F* (-1.35296) reinforce this interpretation, pointing to an increase in rare mutations. However, these values did not reach statistical significance, indicating weak evidence for that interpretation (**Table 1**). In contrast, the strong negative value of Fu’s Fs (-8.71) further supports the notion of population expansion. The significant d_N_/d_S_ ratio of 1.85 (*p* = 0.03) suggests positive selection, where beneficial non-synonymous mutations are favored, indicating adaptive evolution in the population (**Table 2**). The inter-species McDonald-Kreitman (MK) test ratio for PfRh5 was greater than 1, indicating purifying selection, but it did not reach statistical significance (*p* = 0.89) (**Table 2**). Together, these metrics suggest that PfRh5 is experiencing natural selection characterized by recent population expansion and positive selection, where advantageous mutations are proliferating while deleterious mutations are being purged from the population.

**Table 2:** McDonald-Kreitman (MK) test on *pfrh5* with *prrh5* as out-group species and d_N_-d_S_.

	Polymorphic changes within *P. falciparum*		Fixed differences between *P. falciparum* and *P. reichenowi*		Neutrality index (*p* value)^a^	Fu’s Fs	d_N_-d_S_ (*p* value)
	Syn	Non-syn	Syn	Non-syn			
PfRh5	3	9	65	178	1.096 (0.89)	-8.710	1.85 (0.03)

a Fisher’s exact test *p*-value.

**Figure 3:**
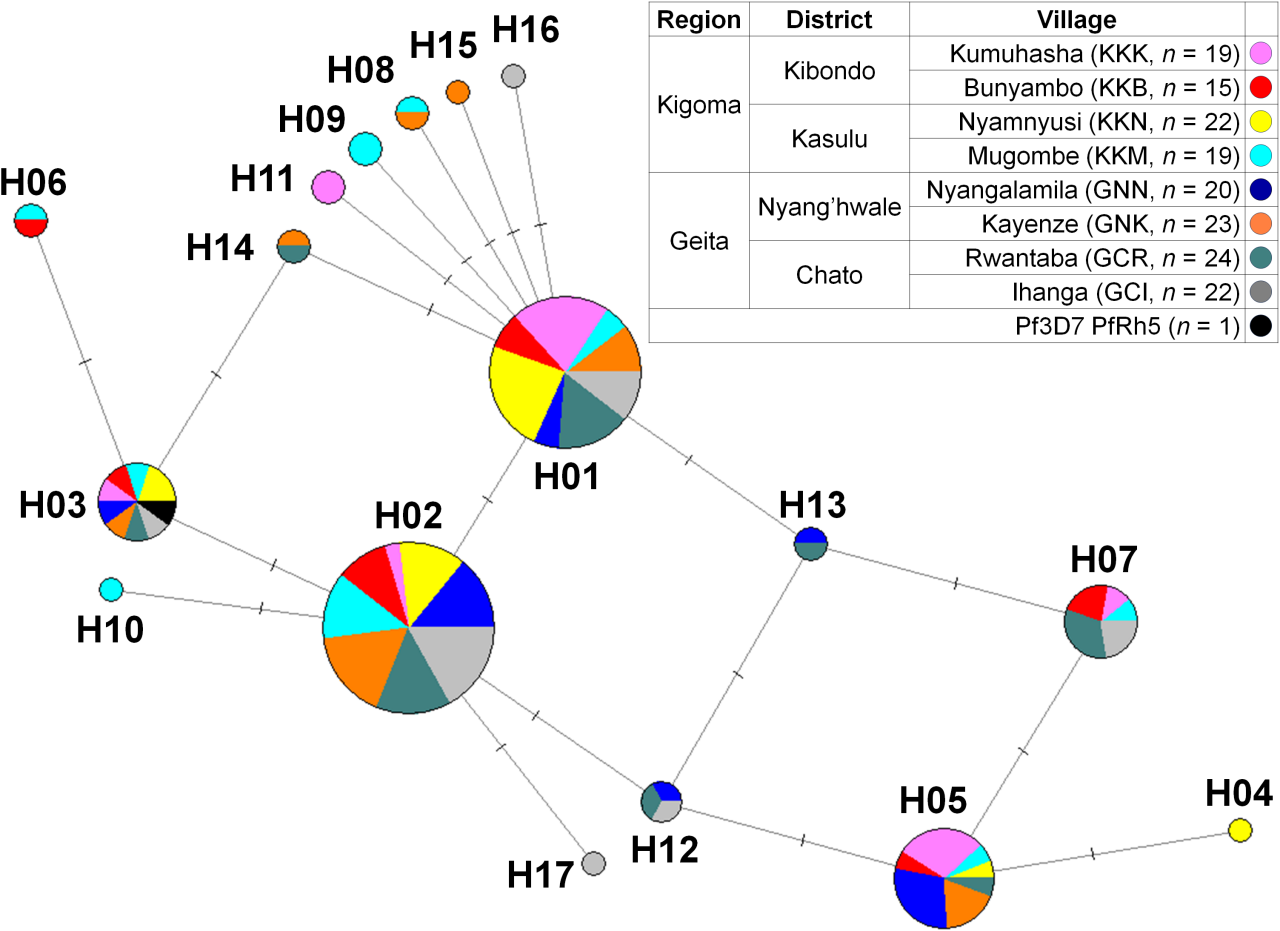
Median-joining networks of PfRh5 haplotype. Geographical haplotype network analysis of PfRh5 was constructed using the NETWORK 10.2 software with the Median Joining algorithm. The network showed 17 haplotypes found in 164 clinical isolates with geographically mixed genotypes.

Geographical haplotype networks of PfRh5 were constructed using the Median-Joining method, revealing a total of 17 haplotypes with mixed geographic patterns (**Figure 3**). The network analysis indicated that PfRh5 underwent population expansion within the study area, without showing a distinct geographical pattern.

### Humoral immune response screening

**Figure 4:**
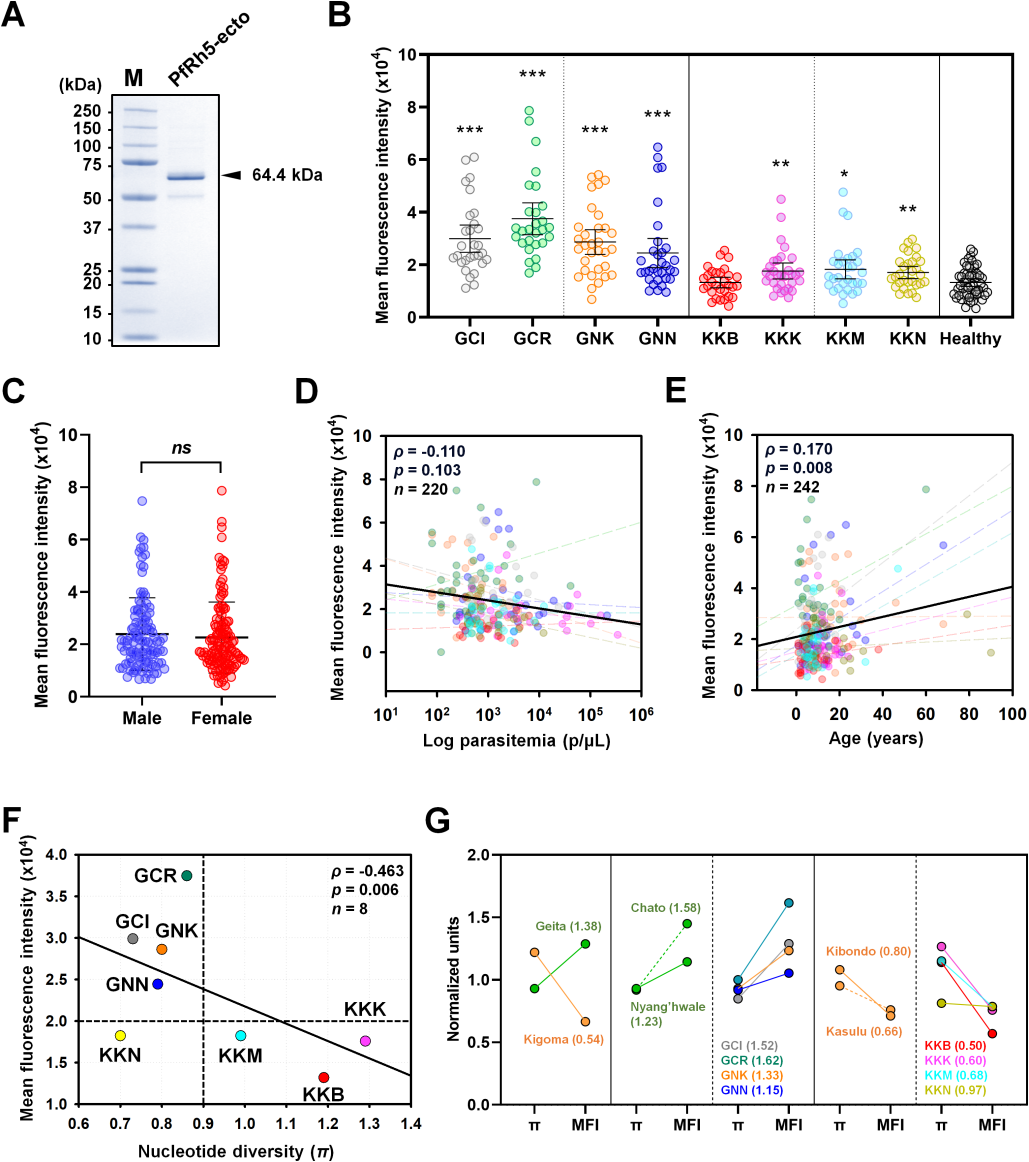
Humoral immune response against PfRh5-ecto. **(A)** Purity confirmation of recombinant PfRh5-ecto (25–526 aa, 64.4 kDa) expression was confirmed by SDS-PAGE. **(B)** Total IgG prevalence was measured in each village with *P. falciparum* patients: GCI (Ihanga), GCR (Rwantaba), GNK (Kayenze), GNN (Nyangalamila), KKB (Bunyambo), KKK (Kumuhasha), KKM (Mugombe), KKN (Nyamnyusi), along with healthy individual sera as a negative control. The scatter plot represents mean fluorescence intensity (MFI) ± 95% confidence intervals (CI). The *p*-values were calculated using unpaired Student’s *t*-test and resulted significance differences are indicated by triple asterisks (*p* < 0.001), double asterisks (*p* < 0.01), and single asterisks (*p* < 0.05). **(C)** Comparison of the total IgG prevalence by gender. ns indicates "not significant". **(D)** The correlation between MFI and individual parasitemia, and **(E)** the correlation with age, were analyzed for each village using Pearson's correlation test (*ρ*). The dots are color-coded by village, as previously described. The dashed lines in different colors represent the correlation trend lines for each village. **(F)** The correlation between total IgG prevalence and nucleotide diversity (*π*) at the village level was shown using Pearson’s correlation test (*ρ*). **(G)** A direct comparison was made between nucleotide diversity (*π*) and total IgG prevalence, calculated using normalized values at each geographical level with fold differences. A fold change greater than 1 indicates a comparatively higher prevalence of IgG relative to nucleotide diversity (*π*), whereas a fold change less than 1 suggests a comparatively lower IgG prevalence.

PfRh5-ecto (25-526 aa.) domains were expressed for antigenicity screening (**Figure 1B**). Recombinant proteins were evaluated for antigenicity across eight villages (**Figure 4A**). Total IgG reactivity was detected in both healthy sera (MFI ± S.D., 13,198 ± 5,556) and asymptomatic patient sera (23,189 ± 13,663) for PfRh5-ecto within the total population. A PfRh5-specific humoral immune response was detected in 34.7% of asymptomatic patients following exposure to *P. falciparum* (**Table 3**). The humoral immune response was higher in patients from Geita (57.9%) region compared to those from Kigoma (11.6%) region (**Figure 4B**; **Table 3**). A direct comparison of the total IgG response by area revealed significant differences between Geita and Kigoma regions (**Supplementary Figure S1B**). Furthermore, when comparing the total population IgG response at the district level (Chato, Nyang’hwale, Kibondo, and Kasulu), variations in antigenicity were observed based on geographical location (**Supplementary Figure S1B**).

**Table 3:** Humoral immune responses against PfRh5-ecto proteins.

Antigen	No. of patient sample			95% CI^b^	MFI^c^	No. of healthy sample			95% CI	MFI	*p* value^e^
	Pos.	Neg.	Total (%)^a^			Pos.	Neg.	Total (%)^d^			
**Total**	**84**	**158**	**242 (34.7)**	**29.0-40.9**	**23,189**	**2**	**54**	**56 (96.4)**	**87.9-99.0**	**13,198**	**< 0.001**
**Geita**	**70**	**51**	**121 (57.9)**	**48.9-66.3**	**29,868**						**< 0.001**
**Nyang’hwale**	**31**	**33**	**64 (48.4)**	**36.6-60.4**	**26,531**						**< 0.001**
Nyangalamila	10	22	32 (31.3)	18.0-48.6	24,437						< 0.001
Kayenze	21	11	32 (65.6)	48.3-79.6	28,625						< 0.001
**Chato**	**39**	**18**	**57 (68.4)**	**55.5-79.0**	**33,614**						**< 0.001**
Rwantaba	24	4	28 (85.7)	68.5-94.3	37,471						< 0.001
Ihanga	15	14	29 (51.7)	34.4-68.6	29,890						< 0.001
**Kigoma**	**14**	**107**	**121 (11.6)**	**7.0-18.5**	**16,510**						**= 0.006**
**Kibondo**	**5**	**56**	**61 (8.2)**	**3.6-17.8**	**15,424**						***ns***
Kumuhasha	4	27	31 (12.9)	5.1-28.9	17,576						= 0.009
Bunyambo	1	29	30 (3.3)	0.6-16.7	13,199						*ns*
**Kasulu**	**9**	**51**	**60 (15.0)**	**8.1-26.1**	**17,614**						**= 0.002**
Nyamnyusi	5	25	30 (16.7)	7.3-33.6	16,996						= 0.008
Mugombe	4	26	30 (13.3)	5.3-29.7	18,231						= 0.012

a Sensitivity: percentage of positive in patient samples.

b CI, confidence interval.

c MFI, mean fluorescence intensity.

d Specificity, percentage of negative in healthy samples.

e Differences in the total IgG prevalence for each antigen between *P. falciparum* patients and healthy individuals were calculated with Student *t*-test. A *p* value of < 0.05 is considered statistically significant. ns indicates "not significant". The bold text indicates the region and district level.

Correlation analysis of PfRh5 humoral immune response by gender showed no significant differences (**Figure 4C**). The parasitemia with antigenicity showed negative correlation, however no significant differences (*ρ* = 0.170, *p* = 0.103) (**Figure 4D**). In contrast, antigenicity showed a significant positive correlation with patient age (*ρ* = 0.170, *p* = 0.008) (**Figure 4E**). The most important factors considered were nucleotide diversity (*π*) and total IgG response. A comparison of the correlation between total IgG response and nucleotide diversity clearly showed a significant negative correlation (*ρ* = -0.463, *p* = 0.006) across different geographical locations (**Figure 4F**). To clearly verify the correlation between genetic polymorphism and antigenicity, we conducted an analysis within regions (**Figure 4G**). For data normalization, we divided each nucleotide diversity (*π*) and mean fluorescence intensity values by the overall mean, normalizing them to “1”. Then, we compared the genetic polymorphism and antigenicity by each region. The results showed that, Geita region had a fold change of mean nucleotide diversity versus antigenicity greater than 1, while the Kigoma region exhibited a fold change of less than 1 (**Figure 4G**). This result indicate that those regions with lower genetic polymorphism maintained higher antigenicity, whereas regions with higher genetic polymorphism displayed lower antigenicity.

## Discussion

*P. falciparum* is genetically diverse because it evolves rapidly, particularly in high transmission areas, to evade the host immune response, adapt to host cells, and overcome interventions such as vaccines and drugs. Genetic diversity in *P. falciparum* is one of the most significant barriers to developing a highly effective malaria vaccine. In malaria high-endemic areas, the simultaneous circulation of genetically diverse strains complicates the assessment of vaccine efficacy. The leading candidates for malaria vaccines, such as CSP for the pre-erythrocytic stage and apical membrane antigen 1 (AMA1) for the blood-stage, exhibit significant genetic diversity across different strains (31, 32). Thus, continuous monitoring of parasite genetic populations is crucial for evaluating the efficacy of vaccine interventions and addressing emerging strains. Strategies such as targeting conserved antigens for developing multi-strain vaccines and incorporating different stages of the parasite’s lifecycle into a single vaccine offer promising solutions to these challenges (33).

The PfRh5 protein plays a crucial role in *P. falciparum* merozoite invasion of erythrocytes through direct interaction with a specific receptor on the host cell surface, known as basigin (CD147) (29). The PfRh5 forms a multi-protein complex that is essential for erythrocyte invasion. This large complex includes CyRPA (Cysteine-rich protective antigen) and Ripr (Rh5-interacting protein). CyRPA interacts with PfRh5 to support its essential function for invasion, while Ripr binds to CyRPA to help stabilize the interaction between PfRh5 and basigin (16). The blood-stage vaccine for *Plasmodium* species focusses on merozoite-blocking strategies, such as those targeting PfRh5, because of its functional importance (13). It is expected to reduce the development of complicated symptoms and provide prevention, even for silent transmission carriers such as asymptomatic cases. One key aspect of PfRh5 is that it is conserved across *P. falciparum* strains, which means that antibodies specific to PfRh5 can effectively block merozoite invasion of erythrocytes (34, 35). These reasons make PfRh5 an attractive target for a blood-stage vaccine. The most recent trial of the PfRh5-based vaccine, which is conducted in Tanzania, was in Phase 1b (20). The results demonstrated that the PfRh5 vaccine was well-tolerated across all age groups and elicited a robust immune response without no serious adverse events, even in younger children and infant (20). This is particularly significant because malaria disproportionately affects young children, who are especially vulnerable to severe disease and death. Furthermore, the vaccine elicited strong immune responses and produced antibodies capable of inhibiting *P. falciparum* invasion of erythrocytes in the laboratory, providing strong evidence for possibility of effective merozoite invasion blocking vaccine.

However, *P. falciparum* vaccine development still faces significant challenges due to genetic polymorphisms. Genetic polymorphisms are a major hurdle for vaccine development as variation can alter the epitope expression, resulting in loss of vaccine efficacy (36). The most advanced *P. falciparum* vaccine candidate is PfCSP, which is an antigen used in the development of the vaccines RTS, S and R21 (37). The C-terminal region of the *pfcsp* gene has been shown to exhibit high genetic diversity in Asia (average π = 0.013), Oceania (0.005), Africa (0.105), and South America (0.018), as well as evidence of balancing selection pressure (38, 39). In comparison, previous studies have shown that PfRh5 has relatively low genetic diversity, with value of 0.0046 in Nigeria and 0.0009 across various regions of Africa, including DRC, Ghana, Guinea, Malawi, Tanzania, and Zambia (31, 40). Our study revealed an average genetic diversity of 0.0008 in Tanzania, with variability at the village level. The average nucleotide diversity results are consistent with previous reports from other regions in Africa (31). However, this study observed rare allele polymorphisms in the study sites for non-synonymous mutations at codons D65H, H170N, I227M, V371I, and I410M. Although these mutations were not found at high frequencies, they provide evidence of differences between geographic regions. Notably, the novel non-synonymous mutation H170N occurred in Kumuhasha (KKK, *n* = 2), and I227M was found in Mugombe (KKM, *n* = 2), suggesting potential active novel mutation pressure in Kigoma. A mutation at amino acid position D65 was reported in Senegal as D65Y at 1.5% (1/65) whereas in Tanzania a different substitution D65H appeared at 1.2% (2/164) in Kayenze (GNK, *n* = 1) and Mugombe (KKM, *n* = 1). Some studies have reported high frequencies of common mutations and low frequencies of region-specific alleles in Mali, Nigeria, Kenya, and even in global isolates (40–43). These results on genetic variation and tests of natural selection support the hypothesis that the genetic evolution of PfRh5 is due to recent population expansion under positive selection pressure. Taken together with the haplotype distribution results at the study site, the recent increase in the rare allele could potentially affect future vaccine efficacy. Some of the mutations were confirmed to not be significantly affected by SNPs in terms of invasion inhibition by antibodies (43). However, continuous monitoring is needed to determine whether the low frequency of SNPs might affect invasion inhibition and vaccine efficacy.

The antigenicity screening results showed no relationship between observed haplotype variation and antigenicity. However, significant differences in antigenicity were found based on geographical location, particularly in Geita (57.4%) and Kigoma (11.6%) regions. This result directly reflects that higher levels of genetic polymorphisms affect lower antigenicity. We expressed recombinant protein based on the Pf3D7 strain (haplotype 3), which may elicit a partial humoral immune response in this high transmission region. It is possible that parasitemia is not a significant factor influencing the PfRh5 immune response, as confirmed by this study and previous reports (44). Despite significantly higher parasitemia levels in Kigoma compared to Geita, the antigenicity remains low. Additionally, correlation analysis indicates a weak relationship between parasitemia and antigenicity. This study also observed that age positively correlates with antigenicity, possibly due to age-dependent exposure to *P. falciparum* in high endemic areas. This finding is consistent with a previous study in Ghanaian children, which demonstrated a short-lived antibody response to PfRh5, a factor that may be relevant in this context (44).

Overall, the analysis of genetic diversity and antigenicity at the village level in high-endemic areas of Tanzania among asymptomatic patients reveals that genetic variation directly affect humoral immune responses. Thus, it is necessary to investigate the impact of various SNPs in a diverse range of field strains, especially in high-endemic areas, which may influence PfRh5 vaccine effectiveness. PfRh5 is the most promising target for a blood-stage vaccine to date. This result highlights the importance of understanding how geographic differences in polymorphisms influence humoral immune responses for merozoite invasion blocking, which might be an important consideration for developing highly effective PfRh5-based vaccines in the future.

## Data Availability

The data presented in the study are deposited in the NCBI GeneBank repository, accession number PQ3374615-PQ337778. (Link: https://www.ncbi.nlm.nih.gov/popset/?term=2813514662).
