# Supplementary material for: Estimation of PfRh5-based vaccine efficacy in asymptomatic Plasmodium falciparum patients from high-endemic areas of Tanzania using genetic and antigenicity variation screening
Source: Front Immunol. 2024 Nov 18;15:1495513. doi: 10.3389/fimmu.2024.1495513 (PMC11609159; doi:10.3389/fimmu.2024.1495513)
Supplement: Supplementary file 1 [file DataSheet1.pdf]

Supplementary file

Estimation of PfRh5-based vaccine efficacy in asymptomatic *Plasmodium falciparum* patients from high-endemic areas of Tanzania using genetic and antigenicity variation screening

Hojong Jun[1], Ernest Mazigo[1,2], Wang-Jong Lee[1], Johnsy Mary Louis[1], Jadidan Hada Syahada[1], Fadhila Fitriana[1], Jin Heo[1], Yeonkyung Kim[1], Boeun Kwon[1], Fauzi Muh[3], Feng Lu[4], Md Atique Ahmed[5], Se Jin Lee[6], Sunghun Na[6], Wanjoo Chun[7], Won Sun Park[8], Min Hong[9], Joon-Hee Han[9], Tae-Hyung Kwon[9], Soo-Ung Lee[9], Eun-Teak Han[1], Jim Todd[10,11], Alphaxard Manjurano[2], Winifrida Kidima[12], and Jin-Hee Han [1,*]

[1] Department of Medical Environmental Biology and Tropical Medicine, School of Medicine, Kangwon National University, Chuncheon, Republic of Korea

[2] Department of Parasitic Diseases, National Institute for Medical Research, Dar es Salaam, Tanzania

[3] Faculty of Public Health, Department of Epidemiology and Tropical Diseases, Universitas Diponegoro, Semarang, Indonesia

[4] Department of Pathogen Biology and Immunology, School of Medicine, Yangzhou University, Yangzhou, China

[5] Malaria Division, Indian Council of Medical Research (ICMR)-Regional Medical Research Centre, Dibrugarh, Assam, India

[6] Department of Obstetrics and Gynecology, Kangwon National University Hospital, Chuncheon, Republic of Korea

[7] Department of Pharmacology, School of Medicine, Kangwon National University, Chuncheon, Republic of Korea

[8] Department of Physiology, School of Medicine, Kangwon National University, Chuncheon, Republic of Korea

[9] Institute of Biological Resources, Chuncheon Bioindustry Foundation, Chuncheon, Republic of Korea

[10] Department of Population Health, London School of Hygiene and Tropical Medicine, London, UK

[11] Department of Biostatistics, Catholic University of Health and Allied Sciences (CUHAS), Mwanza, Tanzania

[12] Department of Zoology, College of Natural and Applied Sciences, University of Dar es Salaam, Dar es Salaam, Tanzania

*Corresponding author

E-mail: han.han@kangwon.ac.kr

Supplement Table S1. Primer information

Gene (position)	Primer sequence
**PCR primer***	F: 5’- cccatgaagaattgagtcacataatagaATGAT-3’
	R: 5’- ccatgttttgtcattTCATTGTGTAAGTGGT-3’
**Sequencing primer 1**	R: 5’-CCACATTTTTATAGTCTTCATTATTTTTCACA-3’
**Sequencing primer 2**	F: 5’-GATGGCATGTTATTAAATGAAAAAAATGAT-3’
**Sequencing primer 3**	F: 5’-GGATATGAAAAATTATGGTACAAACCTTT-3’
**PfRh5-ecto In-fusion cloning****	F: 5’-tcttgga***gcggccgc***cTTCGAGAACGCCATCAAGAAAACC-3’
	R: 5’-taagctt***ggcgcgcc***CTGGGTCAGGGGCTTGT-3’

*, Lowercase letters indicate the 5' or 3' UTRs, while uppercase letters indicate the PfRh5 open reading frame.

**, Italicized and underlined text indicates the restriction enzyme site within the expression vector, and capital letters represent the PfRh5-specific primer site.

Supplement Table S2. Demographical information relevant to *P. falciparum* prevalence

**Region**	**Geita**				**Kigoma**			
**District**	**Chato**		**Nyang'hwale**		**Kibondo**		**Kasulu**	
**Village**	**GCI**	**GCR**	**GNK**	**GNN**	**KKB**	**KKK**	**KKM**	**KKN**
**Total, *n***	29	28	32	32	30	31	30	30
**Age**								
Mean (S.D.)	9.4 (4.9)	9.0 (11.2)	16.3 (13.2)	13.3 (13.0)	8.0 (7.9)	12.2 (5.3)	9.5 (8.8)	16.6 (19.9)
Median	10	6.5	16	9	6	12	8	9
Range	1-21	1-60	1-68	1-68	1-38	3-24	2-47	4-90
**Age group, *n* (%)**								
≤5	7	12	5	10	15	4	9	3
6-10	12	8	7	7	9	7	17	15
11-15	7	5	3	7	3	13	2	4
16-20	2	2	11	1	1	5	0	0
21-25	1	0	2	3	1	2	0	5
25<	0	1	4	4	1	0	2	3
**Gender, *n* (%)**								
Female	16	13	18	22	19	19	12	14
Age, mean (S.D.)	10.1 (5.0)	11.9 (15.4)	16.4 (9.9)	12.7 (8.4)	9.6 (9.1)	12.6 (5.6)	17.5 (23.1)	7.8 (1.9)
Male	13	15	14	10	11	12	18	16
Age, mean (S.D.)	8.6 (4.7)	6.5 (4.8)	16.2 (16.9)	14.6 (20.2)	5.2 (4.4)	11.6 (5.1)	10.6 (11.2)	15.6 (18.2)

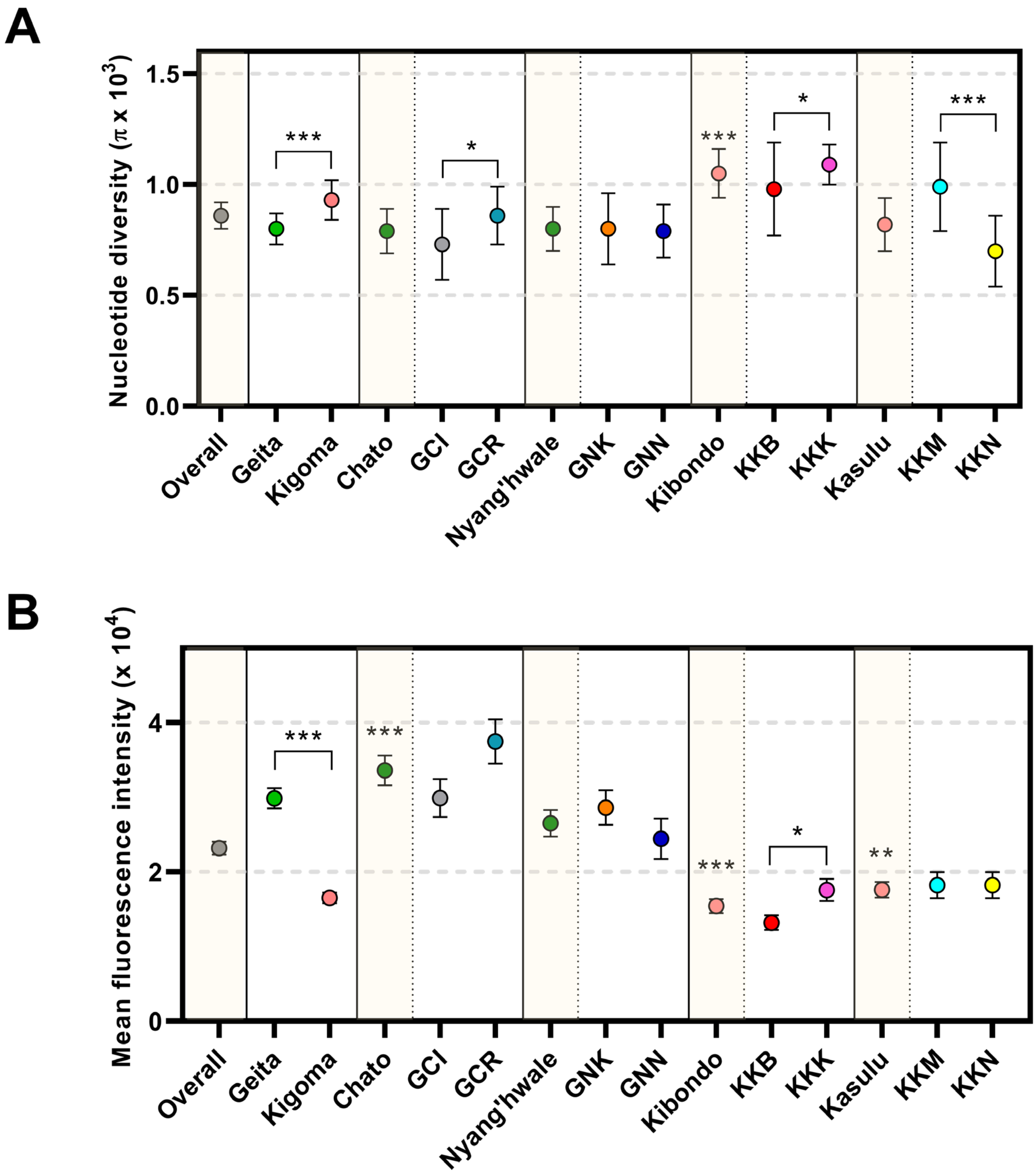

Fig. S1. Statistical analysis of nucleotide diversity (π) and total IgG response at the village, district, and overall levels. (A) Nucleotide diversity (π) and **(B)** total IgG response (mean MFI ± SD) was statistically compared at the regional level (Geita vs. Kigoma) and between villages within each district (e.g., GCI vs. GCR in Chato district). Comparisons at the district level are shown in the district column alongside the overall level. The *p*-values were calculated using unpaired Student's *t*-test, with significant differences indicated by triple asterisks ($p < 0.001$), double asterisks ($p < 0.01$), and single asterisks ($p < 0.05$).
